# Prevalence and Antimicrobial Resistance of Bacterial Foodborne Pathogens Isolated from Raw Bivalve Molluscs Subjected to Consumption in Poland during a Ten-Year Period

**DOI:** 10.3390/foods11213521

**Published:** 2022-11-04

**Authors:** Magdalena Lopatek, Kinga Wieczorek, Jacek Osek

**Affiliations:** Department of Hygiene of Food of Animal Origin, National Veterinary Research Institute, Partyzantow 57, 24-100 Pulawy, Poland

**Keywords:** foodborne pathogens, raw bivalve molluscs, prevalence, antimicrobial resistance

## Abstract

The aim of this study was to evaluate the microbiological contamination of raw bivalve molluscan shellfish (BMS) available on the Polish market and determinate the antimicrobial resistance of the obtained isolates. A total of 1000 mollusc samples were tested for the presence of *Salmonella* spp., *L. monocytogenes*, *V. parahaemolyticus*, and *S. aureus* using the ISO standard methods. Additionally, the bacterial isolates’ susceptibility to antimicrobials was determined using the minimum inhibitory concentration (MIC) method. The obtained results showed that *Salmonella* spp. was detected in 31 (3.1%) samples, and 51.6% of the bacterial isolates were classified as *Salmonella* Typhimurium. A total of 74.2% of the *Salmonella* isolates were sensitive to all antimicrobial agents, whereas three isolates were multiresistant. *L. monocytogenes* was isolated from 18 (1.8%) BMS, and the isolates belonged to serogroups IIa, IIb, and IVb. Most of them were resistant to ceftriaxone (77.8%) and oxacillin (55.6%). *V. parahaemolyticus* was present in 24.2% BMS. These isolates were mainly resistant to ampicillin (77.3%) and streptomycin (64.0%). Moreover, 15.2% of the bivalve molluscs were contaminated with *S. aureus*. Most isolates belonging to this species were resistant to penicillin (84.9%). A total of 60 (6.0%) bivalve molluscs were contaminated with more than one pathogen simultaneously. In addition, the tested bacteria were more likely to be identified during the warmer period (53.9%) compared to the samples analyzed in colder months (35.7%). The obtained results indicate that raw bivalve molluscs from the Polish market are frequently contaminated with bacterial foodborne pathogens, which may be resistant to antimicrobials.

## 1. Introduction

Bivalve molluscs play an important role in the human diet, mainly due to their health benefits, providing a rich source of animal protein, omega-3 fatty acids, vitamin D, selenium, and iodine [1]. The consumption of bivalve molluscs has significantly increased worldwide in recent years, which has led to a high demand for their production and import in various parts of the world [2,3].

BMS can be contaminated with pathogenic microorganisms for humans, including bacteria that are naturally occurring in marine environments, such as *Vibrio* spp., and those associated with water pollution, such as *Salmonella* spp., but also pathogens transmitted to food through contaminated processing environment (*Listeria* spp.) and through poor hand hygiene (coagulase-positive staphylococci, CPS) [3,4]. The presence of bacterial pathogens is often closely correlated with the quality of the waters in which bivalve molluscs live. Household and agricultural wastewater, the uncontrolled storage of organic waste, and municipal and industrial pollution may be sources of the microbiological contamination of foods of marine origin [5,6]. Environmental factors also have a strong influence on the degree of BMS contamination. The variability of temperature, oxygen concentration, and water salinity during that year have a significant impact on the microbial populations in the aquatic environment, as well as on the physiological condition of bivalve molluscs and their ability to filter and accumulate bacteria [7,8,9]. Increases in the water temperature of seas and oceans have been observed as a result of global climate warming, which favors the appearance of pathogenic bacteria in seafood [9,10]. Moreover, bivalve molluscs can be contaminated with some microorganisms after they have been harvested, often due to cross-contamination during processing, distribution, and selling [11]. The greatest risk associated with the consumption of seafood contaminated with bacteria occurs when the bivalve molluscs are consumed raw or after only a short heat treatment. According to the recent EFSA/ECDC report, in European countries in 2020, crustaceans, including raw bivalve molluscs, were the source of 15.3% of confirmed foodborne outbreaks [12].

Bivalve molluscs may also serve as reservoirs of bacteria that are resistant to antimicrobials. The acquisition of a resistance to antimicrobial agents among bacterial pathogens may be a result of their common use in human and veterinary medicine, as well as in agriculture and aquaculture [13,14,15]. An aquatic environment can be a source of antimicrobial-resistant bacteria that can be transmitted directly or with food and water and cause infections in humans, including those that are difficult to treat [13,15]. Therefore, it is very important to conduct studies on the identification of various bacterial pathogens in seafood and their antimicrobial resistance to assess the risk of infection for consumers.

The aim of this study was to evaluate the microbiological contamination of raw bivalve molluscs available on the Polish market and to determine the antimicrobial resistance of the obtained isolates.

## 2. Materials and Methods

### 2.1. Sample Collection

A total of 1000 raw bivalve molluscs were collected during 2009–2018 (100 samples per year) according to the monitoring plan for the bacterial contamination of bivalve molluscs developed by the National Reference Laboratory in Poland. Different species of BMS were examined: clams (n = 437), mussels (n = 269), oysters (n = 225), and scallops (n = 69). All samples, from various countries, were obtained from the wholesale companies and retail shops located all over Poland (Table 1). After collection, the molluscs were immediately transported to the laboratory at a refrigerated temperature and tested within 24 h.

### 2.2. Isolation and Identification of Bacterial Pathogens

The bivalve molluscs (with flesh and intravalvular liquid) were prepared for bacteriological examinations according to the ISO 6887-3:2003 standard [16]. The samples were then homogenized in a blender for 2 min and subjected to specific analyses.

#### 2.2.1. *Salmonella* spp.

*Salmonella* spp. were detected using the ISO 6579:2002 standard method [17]. Briefly, a 25 g sample was pre-enriched in Buffered Peptone Water (BPW, Merck, Darmstadt, Germany) at 37 °C for 18 ± 2 h and then selectively enriched in Muller-Kauffmann Tetrathionate-novobiocin (MKTTn) and Rappaport-Vassiliadis with Soya (RVS) broths (Bio-Rad, Hercules, CA, USA), respectively. Then, one loopful of each both was streaked on two selective media, Xylose Lysine Deoxycholate (XLD) and RAPID’Salmonella agar (Bio-Rad, Hercules, CA, USA), and the suspected bacterial colonies were subjected to biochemical analyses using the API 32E (bioMérieux, Lyon, France) and VITEC 2 Compact systems (bioMérieux, Lyon, France). *Salmonella* isolates were serotyped based on the White–Kauffmann–Le Minor scheme [18] using the relevant polyvalent and monovalent antisera (Sifin Diagnostics, Berlin, Germany; Biomed, Lublin, Poland).

#### 2.2.2. *L. monocytogenes*

Twenty-five grams of bivalve molluscs sample were used for the detection of *L. monocytogenes*, according to the ISO 11290-1:1996 + A1:2004 standard [19]. The method involves selective enrichments in half-Fraser and Fraser broths (bioMérieux, Lyon, France), respectively, followed by the isolation of bacterial colonies on Agar Listeria, according to Ottaviani and Agosti (ALOA, bioMérieux, Lyon, France) and PALCAM agar (Bio-Rad, Hercules, CA, USA). The presumptive *L. monocytogenes* isolates were confirmed by biochemical and CAMP tests, as well as with the API *Listeria* and VITEC 2 Compact systems. Multiplex PCR was used to determine the main *L. monocytogenes* molecular serogroups (IIa, IIb, IIc, and IVb), as described previously [20]. For this purpose, DNA was extracted according to the GenomicMini protocol (A&A Biotechnology, Gdansk, Poland) with modifications by adding 15 μL of lysozyme (10 mg/mL; Sigma-Aldrich, St. Louis, MO, USA) and incubation at 37 °C for 30 min. The DNA amplification was carried out in a thermal cycler (Biometra, Jena, Germany) under the following conditions: initial DNA denaturation at 95 °C for 5 min, followed by 30 cycles of 94 °C for 1 min, 55 °C for 1 min, and 72 °C for 2 min. The final cycle was carried out at 55 °C for 2 min and 72 °C for 5 min.

#### 2.2.3. *V. parahaemolyticus*

The isolation of *V. parahaemolyticus* was performed according to the ISO/TS 21872-1 +Cor1:2008 standard [21]. A 25-g sample was added to Alkaline Saline Peptone Water (ASPW, Bio-Rad, Hercules, CA, USA) used as the first and second enrichment broth. Then, the cultures were plated onto two selective media, Thiosulfate Citrate Bile and Sucrose agar (TCBS, Merck, Darmstadt, Germany) and Tryptone–Soya–Tetrazolium agar (TSAT, in-house, Poland), incubated at 37 °C for 24 ± 2 h. The suspected *V. parahaemolyticus* colonies were first confirmed by the API 32E, VITEC 2 Compact and a halotolerance test with different concentrations of NaCl. Then, PCR for the detection of the species-specific *toxR* gene, which is the recommended method to confirm the presence of *V. parahaemolyticus*, was used [22].

#### 2.2.4. *S. aureus*

*S. aureus* was detected using the ISO 6888-3:2003 + AC:2005 method [23]. The samples were cultured in Giolitti–Cantoni broth (Bio-Rad, Hercules, CA, USA) with potassium tellurite, and the bacterial colonies were isolated on Baird-Parker agar with rabbit plasma and fibrinogen (BP-RPF, bioMérieux, Lyon, France). The obtained coagulase-positive *Staphylococcus* spp. isolates were then confirmed using the API Staph and VITEC 2 Compact for Gram-positive bacteria systems.

### 2.3. Antimicrobial Resistance Testing

One confirmed bacterial isolate from each positive sample was tested for resistance to the respective antimicrobial agents (Table 2). A microbroth dilution method was used to establish the minimum inhibitory concentrations (MICs) of the antimicrobials contained in the appropriate Sensititer custom susceptibility plates (Thermo Fisher Scientific, Waltham, MA, USA) dedicated to each bacterium. Multidrug resistance (MDR) of the tested isolates was defined as resistance to at least three classes of the antimicrobials used in the study (Magiorakos et al. 2012).

#### 2.3.1. *Salmonella* spp.

*Salmonella* isolates were subjected to antimicrobial susceptibility testing against antimicrobials present in the EUVSEC plate (Table 2). The isolates were subcultured twice on nutrient agar, and then, the MIC values were determined with Sensititer Cation Adjusted/TES Mueller–Hinton Broth (Thermo Fisher Scientific) at 36 °C for 18–20 h using the Vision system (Trek Diagnostic System, East Grinstead, UK). The antimicrobials and cut-off values used for the interpretation of the MIC results were in accordance with the European Committee on Antimicrobial Susceptibility Testing (EUCAST) and the European Union Reference Laboratory for Antimicrobial Resistance (EURL-AR) recommendations based on the decision of the European Commission (EC) No. 652/2013 [24] (www.eucast.org; www.eurl-ar.eu; accessed on 20 September 2022).

#### 2.3.2. *L. monocytogenes*

The antimicrobial resistance test for *L. monocytogenes* isolates was performed with the GPN3F plate containing a panel of 17 antimicrobials (Table 2), according to the guidelines of the Clinical and Laboratory Standards Institute (CLSI) [25,26], but the breakpoints for ampicillin, penicillin, and trimethoprim were defined by Lyon et al. [27] and Escolar et al. [28]. Moreover, for the purposes of the present study, isolates marked as intermediate were considered together with isolates sensitive to the antimicrobial agents.

#### 2.3.3. *V. parahaemolyticus*

The MICs of the antimicrobials for the *V. parahaemolyticus* isolates were determined using Mueller–Hinton Broth with 1% NaCl (in-house, Poland) and antimicrobial plates (EUMVS2 and EUVSEC) containing antimicrobials used for the treatment of human infections (Table 2). The obtained results were read using the Vision system and interpreted according to the CLSI guidelines [29]. The breakpoint for streptomycin, which was not established by the CLSI, was taken from earlier studies [30,31].

#### 2.3.4. *S. aureus*

The *S. aureus* isolates were cultured on Columbia agar supplemented with 5% sheep blood (bioMérieux), and the antimicrobial resistance was determined with Sensititer Cation Adjusted/TES Mueller–Hinton Broth and the DKVP and EUST plates. The antimicrobials and cut-off values used to determine the MICs were in accordance with the EUCAST and EURL-AR recommendations (www.eucast.org; www.eurl-ar.eu; accessed on 20 September 2022).

### 2.4. Statistical Analysis

The chi-square test with Yates’s correction was used to the examine the differences in the prevalence of bacterial pathogens and type of bivalve molluscs tested, as well as the distribution of microorganisms using Statistica ver. 10 software (StatSoft, Krakow, Poland). *p* < 0.05 was considered significant.

## 3. Results

### 3.1. Prevalence and Antimicrobial Resistance of Bacterial Pathogens

**Figure 1:**
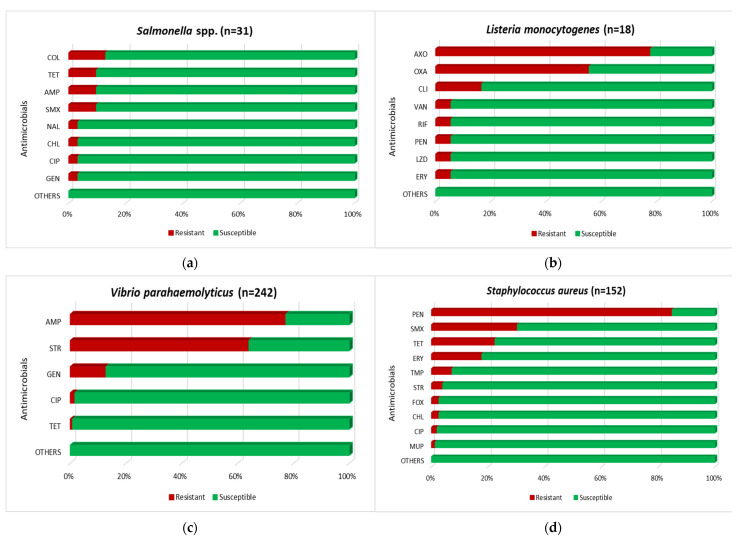
The prevalence (%) of resistance to antimicrobials in tested isolates: (**a**) *Salmonella* spp., (**b**) *L. monocytogenes*, (**c**) *V. parahaemolyticus*, and (**d**) *S. aureus*. COL, colistin; TET, tetracycline; AMP, ampicillin; SMX, sulfamethoxazole; NAL, nalidixic acid; CHL, chloramphenicol; CIP, ciprofloxacin; GEN, gentamicin; AXO, ceftriaxone; OXA, oxacillin; CLI, clindamycin; VAN, vancomycin; RIF, rifampicin; PEN, penicillin; LZD, linezolid; ERY, erythromycin; STR, streptomycin; TMP, trimethoprim; FOX, cefoxitin; MUP, mupirocin.

During the study period, a total of 1000 bivalve molluscs samples were tested for the presence of *Salmonella* spp., *L. monocytogenes*, *V. parahaemolyticus*, and *S. aureus*, and the obtained isolates were evaluated for antimicrobial resistance. The results of these analyses are shown in Table 3, Table S1, and Figure 1, respectively.

#### 3.1.1. *Salmonella* spp.

Thirty-one (3.1%) BMS samples were contaminated with *Salmonella* spp., mainly clams and mussels (13 and 11 samples, respectively). No statistically significant differences (*p* > 0.05) were observed in the presence of this bacterial pathogen in various types of bivalve molluscs. The *Salmonella*-contaminated BMS mostly originated from the Netherlands (15; 48.4% samples) and, to a lesser extent, from other countries (Table 3). Serotyping of the isolates showed that 16 of them (51.6%) belonged to *Salmonella* Typhimurium, including two strains from oysters from the Netherlands, identified as monophasic *S.* Typhimurium (1,4,[5],12:i:-). The remaining 15 (48.4%) isolates were classified as *S.* Enteritidis (two strains), *S.* Branderup (four isolates), *S.* Infantis (four isolates), *S.* Virchow (three isolates), *S*. Agona, and *S.* Derby (one isolate of each).

Most of the tested *Salmonella* isolates (23 out of 31; 74.2%) were sensitive to all antimicrobial agents used, and only a few strains showed resistance to colistin (4; 12.9%), ampicillin, tetracycline, and sulfamethoxazole (3; 9.7% of each) (Figure 1a). Moreover, three *Salmonella* isolates (two from oysters and one from clams, all from the Netherlands), classified as *S.* Typhimurium, displayed multiresistant patterns: AMP-TET-SMX (two strains) and AMP-CHL-CIP-GEN-NAL-SMX (one isolate).

#### 3.1.2. *L. monocytogenes*

*L. monocytogenes* was detected in 18 samples (1.8%), mainly in clams (12; 66.7%), but also in mussels (3 strains) and oysters (3 strains) (*p* > 0.05). Most of the contaminated molluscs originated from the Netherlands and France (six samples of each), and the remaining positive mussels were from Ireland (three samples), Italy (two samples), and Denmark (one sample). Molecular determination of the *L. monocytogenes* serogroups revealed that eight isolates (six from clams from the Netherlands and two from Irish oysters) belonged to serogroup IIa, six isolates (all from clams from France) to IIb, and four isolates (three from mussels and one from oysters from other countries) were classified to the IVb serogroup.

Most *L. monocytogenes* isolates were resistant to ceftriaxone (14 of 18; 77.8%) and oxacillin (10; 55.6%) (Figure 1b). However, only one isolate belonging to molecular serogroup IIa, obtained from clams from the Netherlands, was sensitive to all tested antimicrobials. Moreover, one *L. monocytogenes* strain isolated from French clams and classified in the IIb serogroup was multiresistant to clindamycin, erythromycin, linezolid, oxacillin, penicillin, rifampicin, and vancomycin.

#### 3.1.3. *V. parahaemolyticus*

*V. parahaemolyticus* was identified by the ISO method in 261 (26.1%) samples, whereas PCR for the species-specific *toxR* gene confirmed the presence of this microorganism in 242 (24.2%) samples (Table 3). This number of isolates was used for the subsequent study. The majority of isolates were obtained from clams (151; 62.4%) and mussels (56; 23.1%), whereas the remaining 35 (14.5%) isolates were of oyster and scallop origin (Table 3). Statistically significant differences (*p* < 0.05) were observed when comparing the prevalence of *V. parahaemolyticus* in each type of bivalve molluscs with each other, except a pair of oysters being compared (29 positive samples) and scallops (6 samples) (*p* > 0.05). The greatest differences were observed when the pathogen was in the presence of clams and mussels (*p* = 0.0001), clams and oysters (*p* < 0.0001), and clams and scallops (*p* < 0.0001). Most of the *V. parahaemolyticus*-contaminated molluscs originated from the Netherlands (150; 62.0% samples).

A percentage of *V. parahaemolyticus* isolates was resistant to ampicillin (187; 77.3%) and streptomycin (155; 64.0%) (Figure 1c). In addition, a low number of strains were resistant to gentamicin (31; 12.8%), ciprofloxacin (4; 1.7%), and tetracycline (2; 0.8%). Only four isolates (1.7%), all originating from clams from Italy, were multidrug-resistant, mainly to β-lactams, aminoglycosides, and fluoroquinolones. It was also noted that all analyzed *V. parahaemolyticus* isolates were sensitive to chloramphenicol.

#### 3.1.4. *S. aureus*

The prevalence of *S. aureus* was noted at 15.2% among all tested samples (Table 3). This bacterium was mainly isolated from clams (88 out of 152 positive samples; 57.9%), mussels (39; 25.7%), and oysters (22; 14.5%), usually from the Netherlands (a total of 90; 59.2% contaminated samples). Statistically significant differences were found when comparing the prevalence of this pathogen in clams and oysters (*p* < 0.05), clams and scallops (*p* < 0.05), and also mussels and scallops (*p* < 0.05).

The majority of *S. aureus* isolates were resistant to penicillin (129 out of 152; 84.9%), whereas resistance to sulfamethoxazole, tetracycline, and erythromycin was determined at 30.3%, 22.4%, and 17.7%, respectively (Figure 1d). However, none of the isolates were resistant to gentamicin. The most common antimicrobial resistance profile was PEN-SMX (38; 25.0% isolates), followed by PEN-TET (20; 13.2%), PEN-TET-SMX (9.2%), and ERY-PEN-SMX (6.6%). Moreover, two *S. aureus* strains of clams from Italy and France showed multiresistant patterns on FOX-CHL-ERY-PEN and FOX-ERY-PEN-SMX, respectively.

### 3.2. Simultaneous Occurrence of Bacterial Pathogens

A total of 60 (6.0%) bivalve molluscs were contaminated with more than one pathogen, mainly with two bacterial species simultaneously (59 samples) (Table 4). The majority of these bivalve molluscs were positive for *V. parahaemolyticus* and *S. aureus* (43; 71.6%). The remaining samples were contaminated with *Salmonella* spp. and *S. aureus* (7; 11.7%), *Salmonella* spp., and *V. parahaemolyticus* (5; 8.3%), as well as with *L. monocytogenes* and *V. parahaemolyticus* (4; 6.7%). Interestingly, one sample (a clam from the Netherlands) was positive for three pathogens: *Salmonella* spp., *V. parahaemolyticus*, and *S. aureus* (Table 4). On the other hand, a total of 618 (61.8%) of all samples used in the present study were free from all tested bacterial pathogens, whereas 322 (32.2%) samples were contaminated with only one bacteria, including *Salmonella* spp. (18 samples), *L. monocytogenes* (14 samples), *V. parahaemolyticus* (189 samples), and *S. aureus* (101 samples).

### 3.3. Seasonal Prevalence of Bacterial Pathogens

The present study revealed that the investigated bacteria were more often identified during the warmer period (May–September; 53.9% positive samples) compared to samples analyzed in the colder months (October–April; 35.7%) (*p* < 0.0001) (Table 5). However, this statistical difference was only observed for the prevalence of *V. parahaemolyticus* (*p* < 0.0001), which was detected more often in the warmer period (32.1%) than in the colder months (17.1%).

## 4. Discussion

Bivalve molluscs may be contaminated with bacterial pathogens, and there are serious safety concerns connected with the consumption of raw or undercooked food of marine origin [2,32]. For this reason, studies on the prevalence of pathogenic microorganisms in this kind of food have been performed in many countries. In the present study, the results of the long-term monitoring of the microbiological contamination of raw bivalve molluscs available on the Polish market are described.

One of the microbiological food safety criteria for live bivalve molluscs is the presence of *Salmonella* spp., which must not be present in any of the five analyzed samples [33]. In our study, this microorganism was found in 3.1% of the examined BMS, which makes such food potentially dangerous for consumers. In other investigations, these bacteria were found in 0–2.5% of raw BMS [3,34]. A high prevalence of *Salmonella* spp. was observed in Asian countries, particularly in tropical regions, where this pathogen was detected in 24.5% of the shrimp samples in Vietnam and 34.2% of the clams in India [35,36]. Environmental factors, including water quality, play a significant role in the contamination of foods of marine origin with *Salmonella*, which poses a risk for consumers [3,34,37]. *Salmonella* was isolated from a variety of seafood, such as fish, shrimp, clams, mussels, oysters, crabs, and others, and its prevalence was usually the highest in molluscs, clams, and shrimps [36]. In the current study, although a relatively few positive samples were detected, *Salmonella* was also identified mainly in clams and mussels. Among over 2500 *Salmonella* serovars, only some were connected with food of marine origin, i.e., *S.* Weltevreden, *S.* Rissen, *S.* Derby, and *S.* Typhimurium [36,38]. The latter one was also the most frequently identified during the present study (51.6% of *Salmonella*-positive samples).

*L. monocytogenes* is an important foodborne pathogen responsible for human listeriosis which is especially dangerous disease due to its high mortality rate [3,12]. Only 1.8% of the tested bivalve molluscs samples were contaminated with *L. monocytogenes*. Other surveys have shown that the presence of this pathogen in food of marine origin ranged from 0% (in Greece) to 68.0% (in Denmark) [39,40]. The *Listeria* species most frequently isolated from fish and BMS were *L. innocua*, *L. monocytogenes*, and *L. welshimeri* [40,41]. However, only *L. monocytogenes* is considered the most important cause of food-borne infections and deaths in developed countries [3,42,43]. Most were caused by strains of the IIa, IIb, and IVb serogroups, which were also identified in the present study. It has been described that four *L. monocytogenes* serotypes—1/2a, 1/2b, 1/2c, and 4b classified into the IIa, IIb, IIc, and IVb serogroups, respectively, are typically isolated from approximately 95% of human listeriosis cases [20]. Among them, *L. monocytogenes* serogroup IVb is considered to be the most pathogenic serogroup responsible for the majority of listeriosis outbreaks, whereas serogroup IIa is the most commonly isolated from food [12,20]. The *L. monocytogenes* serogroup IIa was predominant in the bacteria detected in fish in Ireland (>95.0%) and Finland (86.0%), whereas serogroup IVb was revealed only in 14.0% of the isolates [44,45]. Momtaz and Yadollahi [46] reported that *L. monocytogenes* serogroup IVb was most often (66.7%) detected in marine food samples, while serogroups IIa and IIb were identified in 5.6% and 27.8% of bacterial isolates, respectively.

In the investigation, 24.2% of raw bivalve molluscs samples were positive for *V. parahaemolyticus,* and this result is similar to those reported by other authors, where the prevalence of vibrios varied from 0% in BMS from Greece to 35.0% in fish from Portugal [40,47]. The present results indicate that almost all *V. parahaemolyticus* isolates originated from clams, mussels, and oysters, mainly from the Netherlands. In a similar study conducted in Europe, *V. parahaemolyticus* predominantly contaminated oysters (42.2%), mussels (33.1%), and clams (24.7%) [48]. This bacterial pathogen was detected in the marine water of several European countries, including Great Britain, France, Spain, Italy, and Slovenia [2,49]. *V. parahaemolyticus* is often associated with molluscs; however, its high prevalence was also observed in fish, especially in Asian countries [50]. There is no microbiological criterion for raw bivalve molluscs in relation to *V. parahaemolyticus* in the European Union, but testing for the presence of this microorganism is recommended for molluscs harvested from water that was suspected to be contaminated with *Vibrio* sp. [32,40].

In the current study, coagulase-positive *S. aureus* was detected in 15.2% of samples of the examined bivalve molluscs. This is a lower prevalence than was identified in raw bivalve molluscs in Spain (37.0%) and mussels in Greece (56.6%) [40,51]. *S. aureus* is an important bacterial pathogen, causing food-borne infections and intoxications. The most common contamination of the food of marine origin occurs during its processing under poor hygienic conditions. However, for food-borne intoxication, a high number of *S. aureus* is needed. The current microbiological criteria do not provide any requirements for this microorganism [33]. Nevertheless, enterotoxigenic coagulase-positive *S. aureus* may be a serious risk for human health if present in the consumed raw bivalve molluscs [3,40].

The present study shows that some BMS samples (6.0%) were contaminated with more than one tested bacterial pathogens; mainly, they were simultaneously positive for *V. parahaemolyticus* and *S. aureus*. There is little information concerning the prevalence of more than one microorganism in bivalve molluscs. A correlation between the presence of *Salmonella* spp. and *Vibrio* spp. in various kinds of bivalve molluscs was observed by Hariharan and Amadi [15] and Atwill and Jeamsripong [52]. The latter authors noticed that the contamination of shellfish with *Salmonella* spp. was significantly associated with the type and source of molluscs, their sampling location, and the presence of *E. coli* and *Vibrio* spp. [52]. In the current study, it was also found that the presence of more than one pathogen was mainly identified in clams and mussels harvested in the Netherlands (Table 4). However, a relatively low number of these samples did not allow for the determination of any correlation with bivalve molluscs species and their geographical origin.

It was previously shown that the prevalence of bacteria in bivalve molluscs depends on the seasons [8,9,10]. In the present study, *V. parahaemolyticus* was detected more often in the warmer months (32.1% positive samples), when the water temperature usually exceeds 15 °C, than in the colder period (17.1%). The results obtained by Ottaviani et al. [53] showed that this microorganism was identified at a slightly lower level (24.3%) in Adriatic mussels during the warmer months (May–September). Environmental conditions such as salinity and temperature have an important influence on the growth of *V. parahaemolyticus* [38,53,54]. Hatha and Lakshmanaperumalsamy [55] noticed that the lower temperatures during the monsoon months stimulated the growth of *Salmonella* spp. and the prevalence of these bacteria in seafood was significantly higher in the colder period (26.1%) than in the warmer pre- and post-monsoon seasons (6.4 and 7.1%, respectively). Moreover, *L. monocytogenes* can survive and multiply at low temperatures, including marine environments [38].

An antimicrobial resistance analysis of the current bacterial isolates from bivalve molluscs revealed that most *Salmonella* were sensitive to all antimicrobials dedicated to this pathogen and only a few strains showed resistance to colistin, ampicillin, tetracycline, and sulfamethoxazole. The recognized resistance, although referring to a single isolate, has a potential influence on public health because for the treatment of *Salmonella* infections. usually ampicillin, trimethoprim–sulfamethoxazole, and also chloramphenicol were used [56]. However, the crucial is resistance to colistin, which is the antimicrobial last chance in humans for the treatment of multidrug-resistant Gram-negative bacteria. The *mcr* colistin resistance gene may be disseminated between different bacteria because of its presence in the plasmid. It has been shown that many *Salmonella* isolates from various sources possess this gene [57]. In other studies, Gram-negative bacteria isolated from shellfish, including *Salmonella*, were resistant to ampicillin (60.0%), streptomycin (29.0%), amoxicillin–clavulanic acid (20.0%), tetracycline (17.0%), and chloramphenicol (8.6%), and none were resistant to enrofloxacin, ciprofloxacin, and trimethoprim–sulfamethoxazole [15]. In the study of Peruzy et al. [58], 54.0% of *Salmonella* isolates from molluscan shellfish were sensitive to all selected antibacterial agents, whereas 29.0% of the strains of this origin were resistant to one or two antimicrobial classes, and 17.0% were identified as multiresistant isolates.

*L. monocytogenes* is usually sensitive to most antimicrobials used against Gram-positive bacteria, except a natural resistance to the first-generation of quinolones, fosfomycin, and the third-generation of cephalosporins [59,60]. Listeriosis requires effective antimicrobial therapy, which usually includes β-lactam antibiotic (ampicillin or penicillin), sometimes together with gentamicin. The second choice of treatment is a combination of sulfonamide (e.g., sulfamethoxazole) and trimethoprim. In the present investigation, the majority of *L. monocytogenes* isolates were resistant to ceftriaxone and oxacillin, i.e., the antimicrobials that are not clinically important in listeriosis treatment. In a similar study, high resistance rates of these bacteria (isolated from ready-to-eat food, including seafood) to ceftriaxone (49.3%) and oxacillin (90.4%) were also shown [61]. Moreover, in the present investigation, one *L. monocytogenes* isolate was multiantimicrobial-resistant to clindamycin, erythromycin, linezolid, oxacillin, penicillin, rifampicin, and vancomycin, whereas only one isolate of IIa serogroup was sensitive to all the tested antimicrobials.

*Vibrio* spp., including *V. parahaemolyticus*, are usually susceptible to most antibiotics used in veterinary and human medicine. The infection caused by these bacteria usually do not require antimicrobial treatment; however, the antibiotics such as tetracycline or ciprofloxacin are sometimes used in severe or prolonged illnesses [62]. The antimicrobial resistance among the tested *V. parahaemolyticus* revealed that most isolates were resistant to ampicillin and streptomycin. These data are similar to results originating from other countries, where *V. parahaemolyticus* of shellfish origin was mainly resistant to β-lactams (ampicillin) [63,64]. It was also noted that all *V. parahaemolyticus* analyzed in the present study were sensitive to chloramphenicol, whereas other investigations demonstrated that 35–100% of such isolates were resistant to this antimicrobial [63,64].

*S. aureus* is widespread in the environment and in the human body. The resistance of these bacteria to antimicrobials increased worldwide, especially methicillin-resistant *S. aureus* (MRSA), which is clinically important problem, because this group of *S. aureus* is not only resistant to β-lactams but also to many other classes of antimicrobial agents, such as aminoglycosides, quinolones, and macrolides [65]. Nowadays, vancomycin is the drug of choice for treating MRSA infection. Most of the current *S. aureus* isolates showed resistance to penicillin, whereas the levels of resistance to sulfamethoxazole, tetracycline, and erythromycin were much lower. Moreover, none of the *S. aureus* was resistant to gentamicin. In the study conducted by Marijani, [66], among 16 *S. aureus* isolates, 31% and 5% were resistant to tetracycline and gentamicin, respectively. However, a resistance to ciprofloxacin, clindamycin, and penicillin was observed in 19%, 13%, and 13% of isolates, respectively.

Bacteria resistant to antimicrobials occur naturally the environment, including water reservoirs, aquatic plants, and animals. Some substances such as tetracyclines and fluoroquinolones may persist in the environment for months or even years [67]. The resistance-encoding genes can be transferred between the same species but also among not closely related bacteria by horizontal gene transfer of mobile genetic elements. The reservoir of these genes in the environment may be naturally resistant bacteria that also often possess genetic elements carrying multiple traits of resistance.

## 5. Conclusions

In conclusion, the results of the present long-term study investigation indicate that raw bivalve molluscs may be contaminated with various bacterial pathogens, including simultaneous contamination with more than one of the bacterial species. The relatively high prevalence of *V. parahaemolyticus* highlights the importance of raw bivalve molluscs as potential vehicles for transmission of these bacteria. Moreover, this pathogen was detected more often in the warmer period than in the colder months. On the other hand, few molluscan shellfish were contaminated with *L. monocytogenes*, and for 3.1% of the samples, the food safety criterion for *Salmonella* spp. was exceeded. Considering the simultaneous occurrence of bacterial pathogens, it was found that *V. parahaemolyticus* and *S. aureus* were most frequently isolated from bivalve molluscs, mainly clams. Several of these microorganisms were resistant to the commonly used antimicrobials. High levels of resistance to ceftriaxone and oxacillin were observed among *L. monocytogenes* isolates, to ampicillin and streptomycin in *V. parahaemolyticus*, and to penicillin in *S. aureus* isolates, whereas most *Salmonella* spp. were sensitive to all tested antimicrobials. Although, only a few of the bacterial isolates showed multiresistant patterns, continued investigation of the antimicrobial resistance of these pathogens is important to establish effective treatment in humans. To prevent food-borne diseases due to the consumption of raw bivalve molluscs, it would be reasonable to test such food, looking for the bacteria that constitute a potential threat to public health and not only those included in the official microbiological criteria.

## Figures and Tables

**Table 1:** Samples used for microbiological examinations.

Sample Type (Species of Bivalve Molluscs)		Country of Origin	Number of Samples	Total Number (%) of Samples	
Clams	Manila clams	The Netherlands	97	220 (22.0)	437 (43.7)
		Italy	92		
		France	31		
	Razor clams	The Netherlands	78	78 (7.8)	
	Amandes	The Netherlands	41	49 (4.9)	
		France	8		
	Cockle	The Netherlands	40	47 (4.7)	
		France	7		
	Hard clams	Canada	16	43 (4.3)	
		The Netherlands	12		
		France	12		
		Italy	3		
Mussels		The Netherlands	114	269 (26.9)	
		Norway	81		
		Denmark	47		
		Spain	15		
		France	6		
		Italy	4		
		Ireland	2		
Oysters		The Netherlands	166	225 (22.5)	
		France	52		
		Ireland	7		
Scallops		Norway	41	69 (6.9)	
		The Netherlands	18		
		France	4		
		USA	4		
		Italy	2		
All samples				1000	

**Table 2:** Antimicrobials used for the determination of resistance of the tested bacteria.

Antimicrobials	Abbreviation	Application for Resistance Testing			
		*Salmonella* spp.	*L. monocytogenes*	*V. parahaemolyticus*	*S. aureus*
Ampicillin	AMP	+	+	+	−
Penicillin	PEN	−	+	−	+
Oxacillin	OXA	−	+	−	−
Ceftriaxone	AXO	−	+	−	−
Cefotaxime	FOT	+	−	−	−
Cefoxitin	FOX	−	−	−	+
Ceftazidime	TAZ	+	−	−	−
Meropenem	MERO	+	−	−	−
Chloramphenicol	CHL	+	−	+	+
Ciprofloxacin	CIP	+	+	+	+
Nalidixic acid	NAL	+	−	−	−
Gentamicin	GEN	+	+	+	+
Streptomycin	STR	−	+	+	+
Erythromycin	ERY	−	+	−	+
Gatifloxacin	GAT	−	+	−	−
Levofloxacin	LEVO	−	+	−	−
Mupirocin	MUP	−	−	−	+
Linezolid	LZD	−	+	−	−
Clindamycin	CLI	−	+	−	+
Colistin	COL	+	−	−	−
Vancomycin	VAN	−	+	−	+
Rifampicin	RIF	−	+	−	+
Tetracycline	TET	+	+	+	+
Sulfamethoxazole	SMX	+	−	−	+
Trimethoprim	TMP	+	−	−	+
Quinupristin/Dalfopristin	SYN	−	+	−	−
Trimethoprim/Sulfamethoxazole	SXT	−	+	−	−

+, antimicrobial was used; −, antimicrobial was not used.

**Table 3:** Prevalence of bacterial pathogens in the different types of bivalve molluscs tested.

Source		No. (%) of Samples Tested	No. (%) of Positive Samples for			
Sample Type	Country of Origin		*Salmonella* spp.	*L. monocytogenes*	*V. parahaemolyticus*	*S. aureus*
Clams	The Netherlands	268	7	4	92	58
	Italy	95	6	2	42	19
	France	58	0	6	11	11
	Canada	16	0	0	6	0
	Total (%)	437 (43.7)	13 (3.0)	12 (2.7)	151 (34.6)	88 (20.1)
Mussels	The Netherlands	114	2	2	29	13
	Norway	81	5	0	9	13
	Denmark	47	1	1	16	11
	Spain	15	0	0	1	0
	France	6	3	0	0	1
	Italy	4	0	0	1	0
	Ireland	2	0	0	0	1
	Total (%)	269 (26.9)	11 (4.1)	3 (1.1)	56 (20.8)	39 (14.5)
Oysters	The Netherlands	166	5	0	26	18
	France	52	1	0	2	4
	Ireland	7	0	3	1	0
	Total (%)	225 (22.5)	6 (2.7)	3 (1.3)	29 (12.9)	22 (9.8)
Scallops	Norway	41	0	0	3	1
	The Netherlands	18	1	0	3	1
	France	4	0	0	0	0
	USA	4	0	0	0	0
	Italy	2	0	0	0	1
	Total (%)	69 (6.9)	1 (1.4)	0	6 (8.7)	3 (4.3)
All samples (%)		1000 (100)	31 (3.1)	18 (1.8)	242 (24.2)	152 (15.2)

**Table 4:** Simultaneous occurrence of bacterial pathogens in bivalve molluscs samples (n = 60).

Bacterial Pathogens	No. of Positive Samples		
	Sample Type (No. of Samples)	Country Origin (No. of Samples)	Total (%)
*V. parahaemolyticus* + *S. aureus*	clams (33), mussels (7), oysters (3)	the Netherlands (28), Italy (8), Denmark (4), France (3)	43 (71.6)
*Salmonella* spp. + *V. parahaemolyticus*	clams (4), mussels (1)	the Netherlands (3), Denmark (1), Italy (1)	5 (8.3)
*L. monocytogenes* + *V. parahaemolyticus*	clams (2), mussels (1), oysters (1)	the Netherlands (2), France (1), Ireland (1)	4 (6.7)
*Salmonella* spp. + *S. aureus*	mussels (3), clams (2), oysters (2)	the Netherlands (2), Norway (2), Italy (2), France (1)	7 (11.7)
*Salmonella* spp. + *V. parahaemolyticus* + *S. aureus*	clams (1)	the Netherlands (1)	1 (1.7)

**Table 5:** Seasonal distribution of bacterial pathogens in bivalve molluscs tested during 2009–2018.

Sampling	No. of Samples		No. (%) of Positive Samples for Pathogens			
Period	Tested	Positive (%)	*Salmonella* spp.	*L. monocytogenes*	*V. parahaemolyticus*	*S. aureus*
warmer months (May–September)	473	255 (53.9%)	10 (2.1)	12 (2.5)	152 (32.1)	81 (17.1)
colder months (October–April)	527	188 (35.7%)	21 (4.0)	6 (1.1)	90 (17.1)	71 (13.5)

## Data Availability

Data are contained within the article.

## Supplementary Materials

The following supporting information can be downloaded at: https://www.mdpi.com/article/10.3390/foods11213521/s1: Table S1: The prevalence of bacterial pathogens in particular years.
