# Supplementary material for: Prevalence and Antimicrobial Resistance of Bacterial Foodborne Pathogens Isolated from Raw Bivalve Molluscs Subjected to Consumption in Poland during a Ten-Year Period
Source: Foods. 2022 Nov 4;11(21):3521. doi: 10.3390/foods11213521 (PMC9657009; doi:10.3390/foods11213521)
Supplement: Supplementary file 1 [file foods-11-03521-s001.zip › foods-1966672-sm.pdf]

foods

Table S1. The prevalence of bacterial pathogens in particular years

Sampling Year	No. of Tested Samples	No. (%) of Positive Samples For			
		Salmonella spp.	*L. monocytogenes*	*V. parahaemolyticus*	*S. aureuss*
2009	100	4 (4.0)	0	27 (27.0)	22 (22.0)
2010	100	0	0	11 (11.0)	16 (16.0)
2011	100	0	4 (4.0)	17 (17.0)	9 (9.0)
2012	100	2 (2.0)	0	16 (16.0)	10 (10.0)
2013	100	8 (8.0)	2 (2.0)	20 (20.0)	15 (15.0)
2014	100	11 (11.0)	6 (6.0)	26 (26.0)	20 (20.0)
2015	100	1 (1.0)	0	37 (37.0)	19 (19.0)
2016	100	1 (1.0)	2 (2.0)	32 (32.0)	11 (11.0)
2017	100	4 (4.0)	2 (2.0)	36 (36.0)	24 (24.0)
2018	100	0	2 (2.0)	20 (20.0)	6 (6.0)
Total (%)	1,000 (100)	31 (3.1)	18 (1.8)	242 (24.2)	152 (15.2)
